# Supplementary material for: Identification of an RNA Silencing Suppressor Encoded by a Symptomless Fungal Hypovirus, Cryphonectria Hypovirus 4
Source: Biology (Basel). 2021 Jan 31;10(2):100. doi: 10.3390/biology10020100 (PMC7912522; doi:10.3390/biology10020100)
Supplement: Supplementary file 1 [file biology-10-00100-s001.zip › supplementary data.pdf]

Supplementary Data for

Identification of an RNA silencing suppressor encoded by a symptomless fungal hypovirus, Cryphonectria hypovirus 4

Annisa Aulia[a, b], Kiwamu Hyodo[a], Sakae Hisano[a], Hideki Kondo[a], Bradley I. Hillman[c], and Nobuhiro Suzuki[a]

[a] Institute of Plant Science and Resources (IPSR), Okayama University, Kurashiki, 710-0046 Japan

[b] Graduate School of Environmental and Life Science, Okayama University, Okayama, 700-8530 Japan

[c] Plant Biology and Pathology, Rutgers University, New Brunswick, NJ 08901, USA

Running title: CHV4 RNA silencing suppressor

Corresponding author:

Nobuhiro Suzuki
e-mail: nsuzuki@okayama.u-ac.jp
Tel. +82(086) 434-1230
Fax. +82(086) 434-1232

Fig. S1. CHV4-mediated suppression of the GFP reporter induction. The reporter fungal strain with pCPX-C18-*dcl2*pro-eGFP (C18/*dcl2*pro-eGFP) was singly or dually infected by CHV1-Δp69 and MyRV2 (B), or CHV4. A different set of fungal strains than the ones shown inf Figure 1B were used. Values in the respective panels show relative intensity of the reporter eGFP green fluorescence quantified by ImageJ, with the CHV1-Δp69-infected strain expressed as 100.

Fig. S2. The 5′-terminal nucleotide profile of viral-derived small RNA (15- to 32 nt) of *C. parasitica* C18 strain in either singly or doubly infected by CHV4-C18 and MyRV2. Red, dark yellow, blue and green regions show small RNAs with 5'-terminal nucleotides U, G, C, and A, respectively.

Fig. S3. Quantitative analyses of *dcl2* or MyRV2 S4 mRNA in C18 transformed by the empty vector and p24 coding domain. different fungal strains. Bar graphs show the relative accumulation levels (mean ± SD, n = 2) of *dcl2* mRNA or MyRV2 S10 mRNA was quantified by RT-qPCR. Tested fungal strains included C18 transformants with the empty vector (C18emp) and p24 coding domain (C18p24) that were uninfected (virus free) or infected by MyRV2. In RT-qPCR, glyceraldehyde-3-phosphate dehydrogenase gene (gpd) mRNA was used as an internal control. The primers used are: MyRV2 S4 F3 (5'-ACGCGCTTCTGTGATGAATG-3') and MyRV2 S4 R3 (5'-ATCTGTCGCAACAACGGATG-3') for MyRV S4 mRNA, DCL2 F3 (5'-ACGCGAAATCACATCTGCAG-3') and DCL2 R3 (5'-AGCAACACGGTAGCTTTCAG-3') for *dcl2* mRNA, and DCL2 F3 (5'-AAGGGGCAGCGAAGAAAAG-3') and DCL2 R3 (5'-ACGTAGCTAACTACCACTGCAC-3') for *gpd* mRNA. The mean value for MyRV2 C18 wt was set to 100.

Fig. S4. Quantification of *dcl2* or MyRV2 S10 mRNA in different fungal strains. Bar graphs show the relative accumulation levels (mean ± SD, n = 2–5) of *dcl2* or MyRV2 S10 mRNA based on the northern blotting data shown in Figure 4. Tested fungal strains included C18p24 and C18 wt uninfected or infected by MyRV2 alone or together with CHV4-C18. The mean value for MyRV2 C18 wt was set to 100. Different letters indicate significant differences (P < 0.05), by one-way analysis of variance (ANOVA) followed

by Tukey's honestly significant difference (HSD) test. Statistical analyses were conducted with the open source software OpenStat (http:// statpages.info/miller/OpenStatMain.htm).

Fig. S1

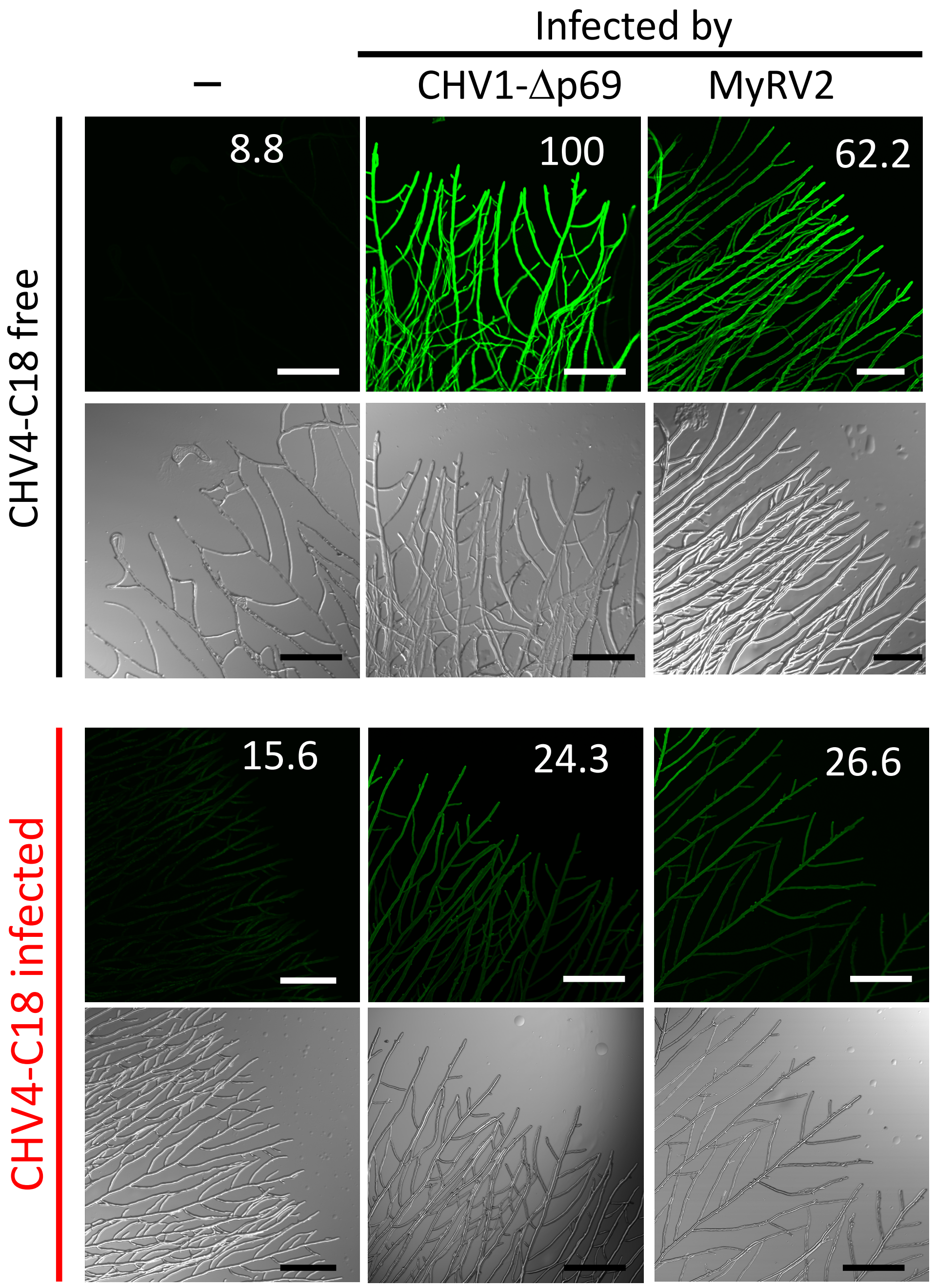

Infected by
–
CHV1-Δp69
MyRV2
CHV4-C18 free
CHV4-C18 infected
8.8
100
62.2
15.6
24.3
26.6

Fig. S2

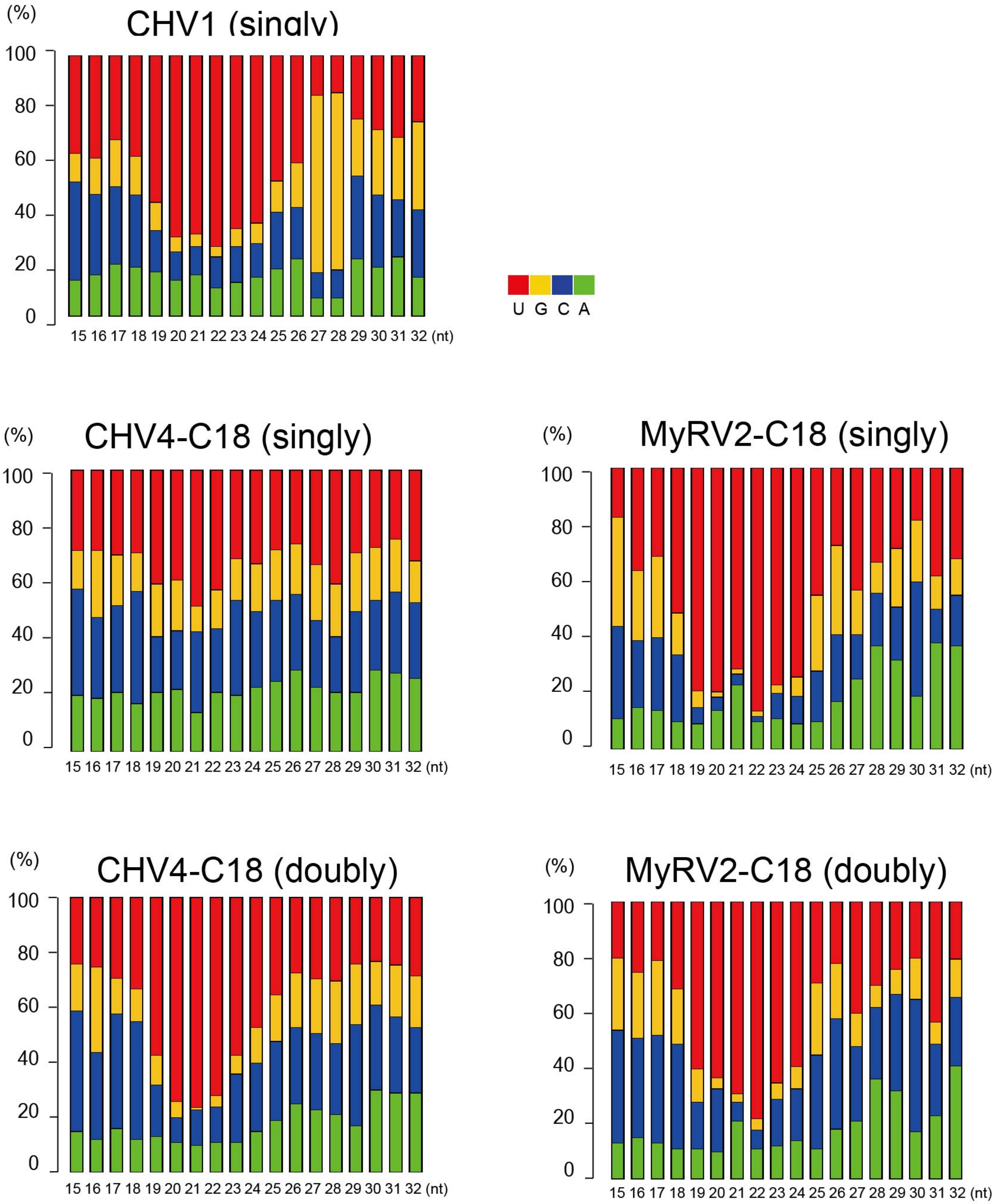

CHV1 (singly)
CHV4-C18 (singly)
MyRV2-C18 (singly)
CHV4-C18 (doubly)
MyRV2-C18 (doubly)
(%)
0 20 40 60 80 100
15 16 17 18 19 20 21 22 23 24 25 26 27 28 29 30 31 32 (nt)
U G C A

Fig. S3

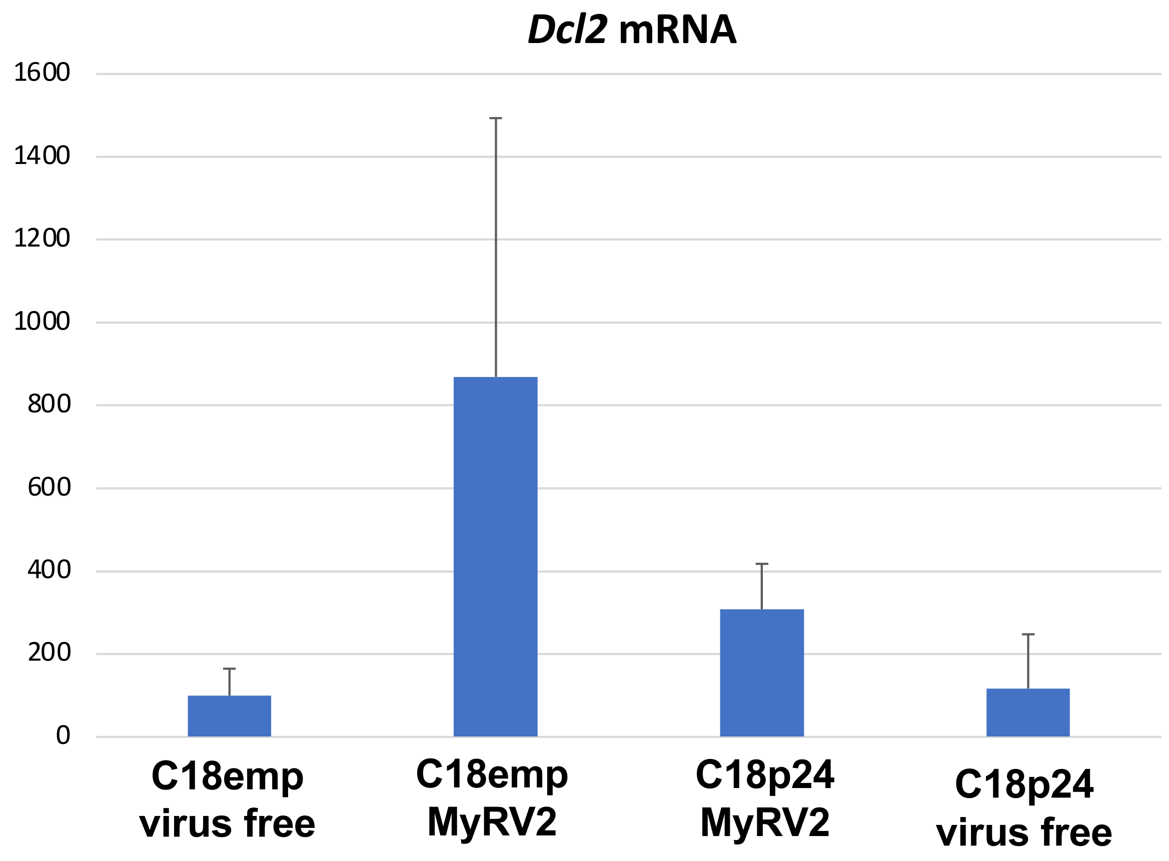

Dcl2 mRNA
1600
1400
1200
1000
800
600
400
200
0
C18emp virus free
C18emp MyRV2
C18p24 MyRV2
C18p24 virus free

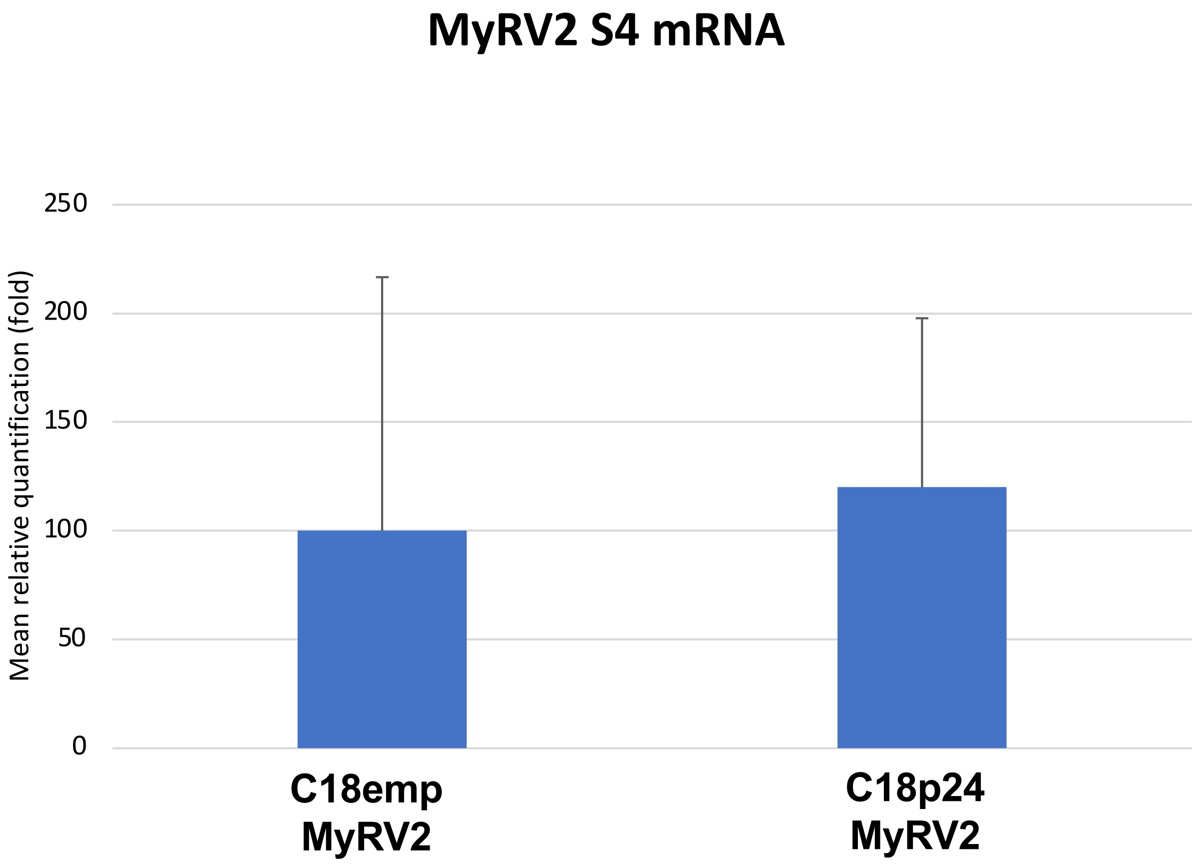

MyRV2 S4 mRNA
Mean relative quantification (fold)
250
200
150
100
50
0
C18emp MyRV2
C18p24 MyRV2

Fig. S4

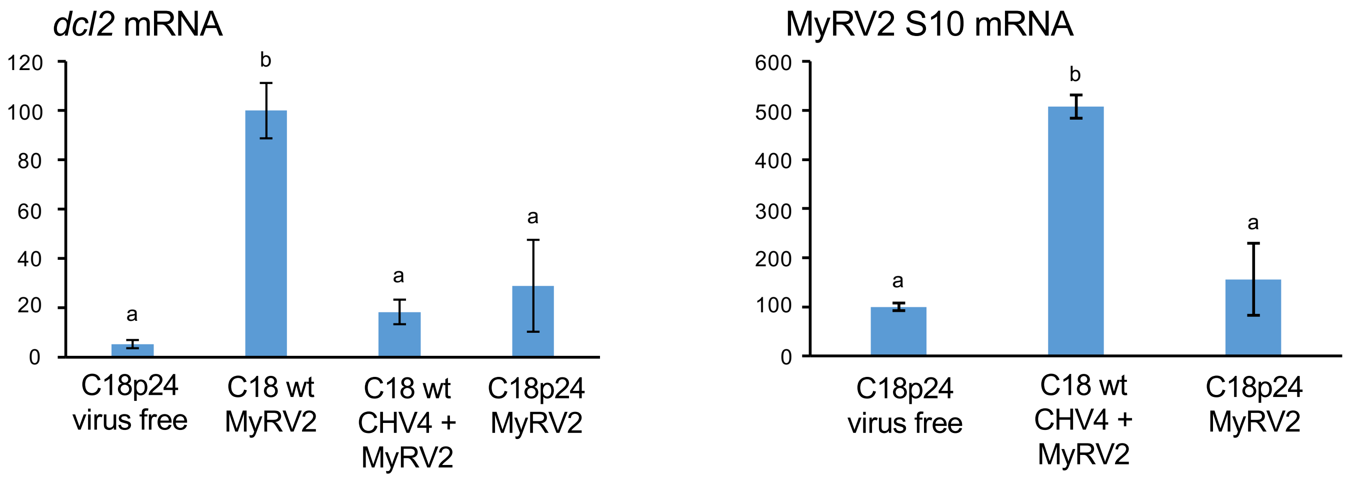

dcl2 mRNA
120
100
80
60
40
20
0
a
b
a
a
C18p24 virus free
C18 wt MyRV2
C18 wt CHV4 + MyRV2
C18p24 MyRV2
MyRV2 S10 mRNA
600
500
400
300
200
100
0
a
b
a
C18p24 virus free
C18 wt CHV4 + MyRV2
C18p24 MyRV2

Supplementary Table S1. List of primers used in the study

Primer Name	Sequence (5′-3′)	Direction	Remarks
NotI C18DCL2Pro R	GCATGCGCGGCCGCCTTGCAGCGTCGTACGACAGAT	Reverse	C18 *dcl2pro::egfp*
SalI C18DCL2Pro F	CCCCCTGTCGACTGAGGAGGGTGGGGACAAAGGT	Forward	
NotI eGFP F	GTTAACGCGGCCGCATGGTGAGCAAGGGCGAGGAGC	Forward	C18 *dcl2pro::egfp* and egfp probe
SphI eGFP R	AGGTCAGCATGCTTACTTGTACAGCTCGTCCATG	Reverse	
CHV4-287 F	ATGTCTGAGCAACAACTCATCT	Forward	CHV4 RT-PCR
CHV4-853 R	TGCCATCCACCAGATGCCAGTT	Reverse	CHV4 RT-PCR
DCL2_4025_F	CCTGCCCTGTTCAGTATCA	Forward	C18 dcl2 KO
DCL2_4545_R	GTGGTAGCCCTCTCTTTGAC	Reverse	C18 dcl2 KO
pCold-I-KpnI-CHV4P52_F	CCCATATGGAGCTCGGTACCATGTCTGAGCAACAACTCATCTA	Forward	CHV4 protease cleavage assay
pCold_NdeI-CHV4-p52_F	ATATCGAAGGTAGGCATATGTCTGAGCAACAACTCATCTA	Reverse	CHV4 protease cleavage assay
pCPXHY3-HpaI-CHV4p24_F	ACGCGGCCAAGCTTGTTAACATGTCTGAGCAACAACTCATCTA	Reverse	C18 p24
pCPXHY3-HpaI-CHV4P24_R	ATGCGCGGCCGCGTTAACCCAAGGCGTGCACGCTTTGTC	Reverse	C18 p24
MyRV2-E2-10_300_F	AATTCAATTCCGCGCGAAGGGG	Forward	MyRV2 S10 probe
MyRV2-E2-10_1200_R	TTCATTTTCACGTTGTTAAAAC	Reverse	MyRV2 S10 probe
5'UTR_CHV1_F	GATAATTTTGGTTGCTGCAC	Forward	CHV1-Δp69 RT-PCR and probe
5'UTR_CHV1_R	GACTCATGTGGCGACGTGCC	Reverse	CHV1-Δp69 RT-PCR and probe
