# Supplementary figures and images for: Identification of an RNA Silencing Suppressor Encoded by a Symptomless Fungal Hypovirus, Cryphonectria Hypovirus 4
Source: Biology (Basel). 2021 Jan 31;10(2):100. doi: 10.3390/biology10020100 (PMC7912522; doi:10.3390/biology10020100)

Figure 1C

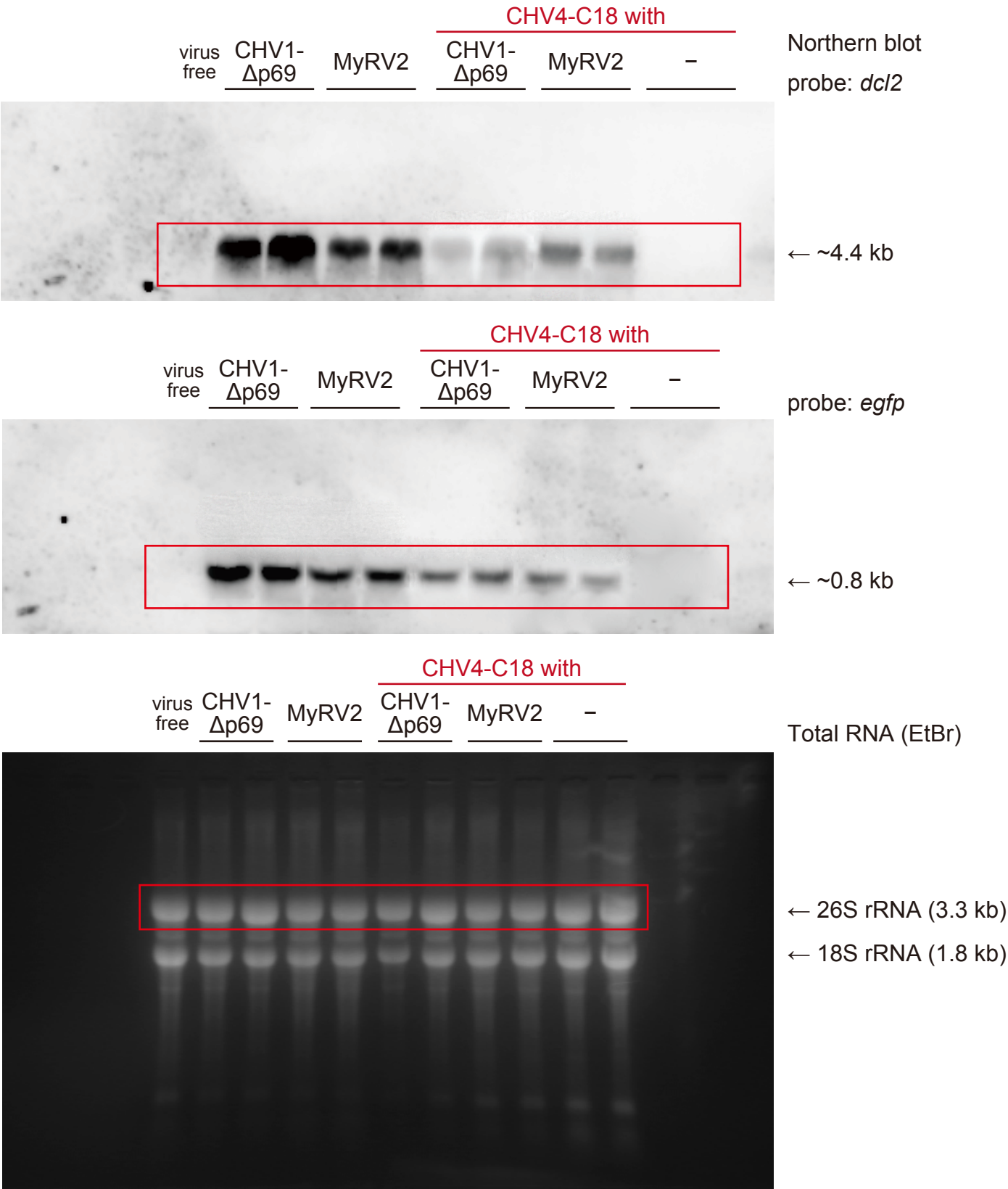

Figure 4

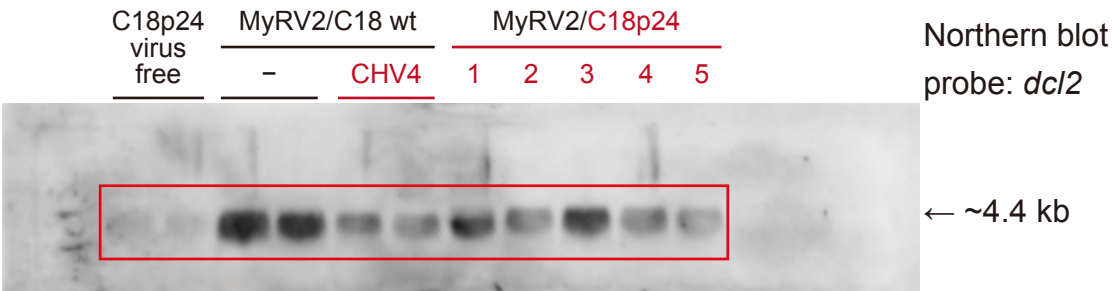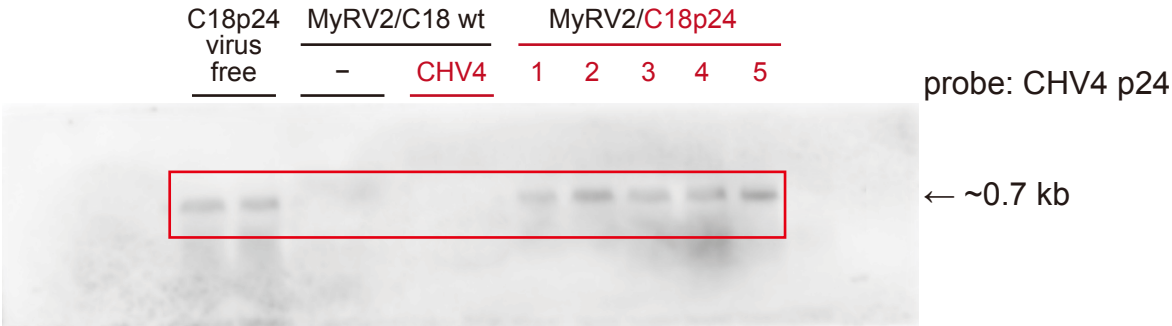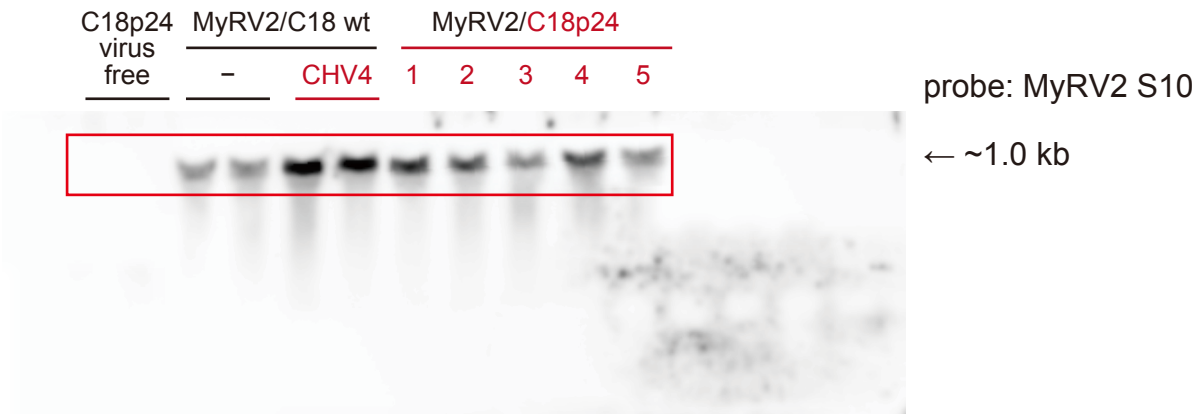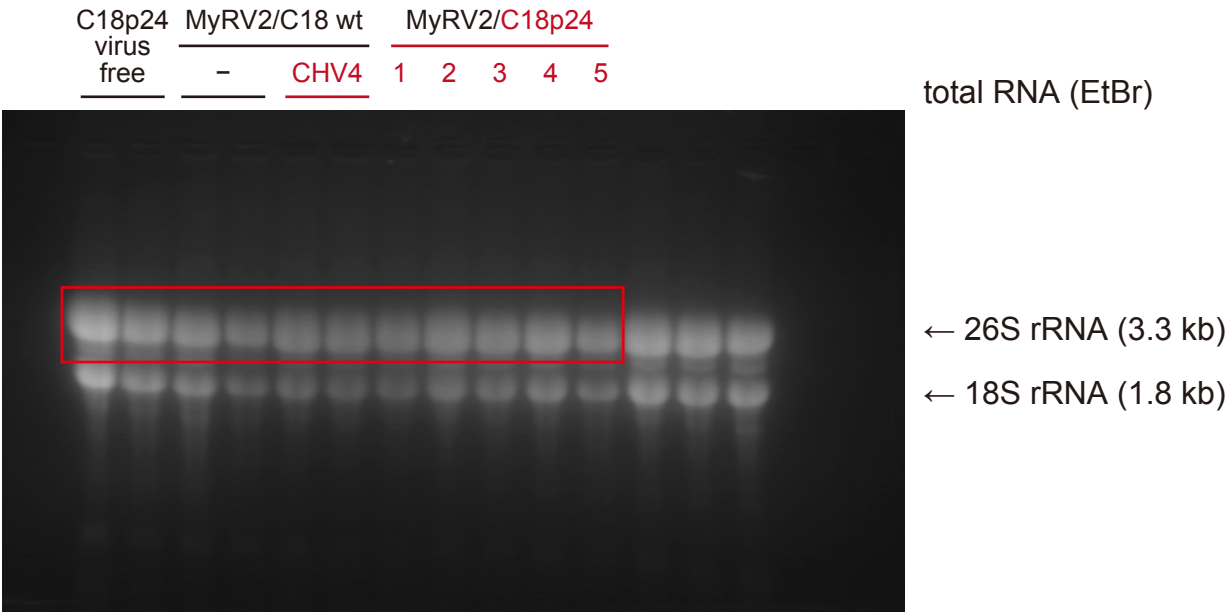

Supplement: Supplementary file 1 [file biology-10-00100-s001.zip › biology-1096190-WB.pdf]
